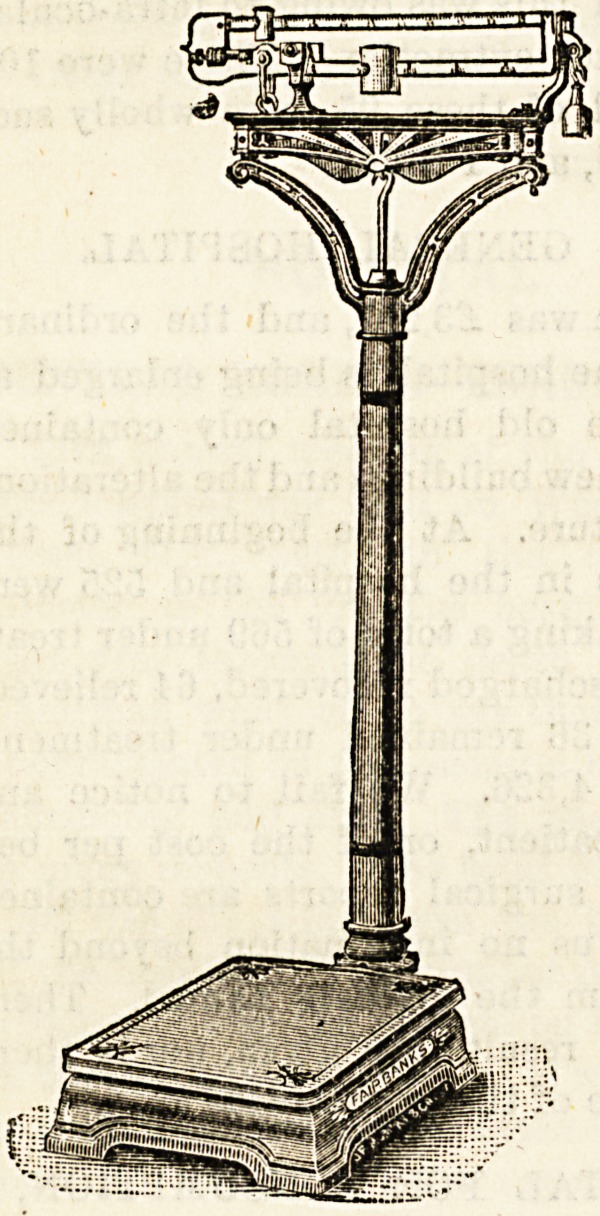# Practical Departments

**Published:** 1903-07-04

**Authors:** 


### PRACTICAL DEPARTMENTS.

### PERSONAL WEIGHING MACHINES.

The accompanying block shows a weighing machine designed for use in physicians' consulting-rooms, hospitals, etc. It will weigh up to 25 stone by means of an extra 5-stone weight, or 150 kilograms by 100-grain divisions. It is unnecessary to stoop to read the figures registered, owing to the convenient height of the pillar, and there are no loose weights except the one just mentioned. The scale is of metal throughout, finished in black or white enamel, with gold tracing, and the sliding poise steelyard is nickel-plated. The platform measures $13\frac{1}{2}$ by $19\frac{1}{2}$ inches. A machine, of which the platform is somewhat smaller, is constructed for use in bathrooms, for which the small space it occupies renders it specially suitable. It is finished with blue-tinted white enamel, and can be graduated in the metric system to 125 kilograms, by 20-gram divisions. Either machine can be fitted with a measuring rod. A removable chair of solid oak, finely polished and upholstered in leather, is made for use with a machine which runs on castors. It is of 32-stone capacity. All the above, with other varieties, are manufactured by the Fairbank Company, 78–80 City Road, London, E.C. A limited number of weight-charts will be supplied by the firm to any hospital applying for them.

## Figures and Tables

**Figure f1:**